# Association of the new TyG indicator–TyHGB with prediabetes and diabetes in middle-aged and elderly postmenopausal women: a longitudinal study

**DOI:** 10.3389/fendo.2026.1871848

**Published:** 2026-07-03

**Authors:** Ziran Xiu, Zhengnan Gao, Lan Luo, Peiyang Yu

**Affiliations:** 1 Clinical Skills Training Center, Central Hospital of Dalian University of Technology (Dalian Municipal Central Hospital), Dalian, China; 2 Department of Endocrinology and Metabolism, Central Hospital of Dalian University of Technology (Dalian Municipal Central Hospital), Dalian, China; 3 Department of Urology, Central Hospital of Dalian University of Technology (Dalian Municipal Central Hospital), Dalian, China

**Keywords:** diabetes, longitudinal study, postmenopausal women, prediabetes, TyHGB

## Abstract

**Introduction:**

Given the established relationship between lipid metabolism and dysglycemia, this longitudinal study aimed to investigate the association between the triglyceride-high density lipoprotein cholesterol-glucose body index (TyHGB) and the risk of developing prediabetes or diabetes among middle-aged and elderly postmenopausal women.

**Methods:**

Data were collected from the REACTION study in Dalian, China. The study included postmenopausal women with normal baseline glucose tolerance who were followed up over a three-year period. We used multivariable logistic regression to evaluate the relationship of TyHGB with incident prediabetes and diabetes. Restricted cubic splines (RCS) were employed to examine dose-response relationships. The predictive performance of TyHGB and traditional indices for prediabetes and diabetes was evaluated and compared using receiver operating characteristic (ROC) curve analysis.

**Results:**

Among the 2,138 postmenopausal women with normal glucose levels at baseline, 598 developed prediabetes and 124 developed diabetes during the follow-up period. Multivariable logistic regression revealed a significant positive association between TyHGB and the risk of both prediabetes and diabetes. Restricted cubic spline analysis confirmed a nonlinear dose-response relationship, with prediabetes risk increasing steeply below an inflection point of 8.52 before plateauing, whereas diabetes risk remained stable below 6.47 and increased sharply thereafter. Subgroup analyses demonstrated that this association remained consistent across strata defined by age, hypertension, hyperlipidemia, and coronary heart disease (CHD). Additional ROC curve analyses suggested that TyHGB may provide effective predictive value for diabetes in postmenopausal women.

**Conclusion:**

In a middle-aged and elderly postmenopausal population, the TyHGB index is a significant independent risk factor for prediabetes and diabetes.

## Introduction

Diabetes mellitus affects approximately 451 million individuals worldwide, with a steadily increasing prevalence across countries of various income levels (1). It is estimated that by 2045, the number of individuals with prediabetes will exceed 600 million (2), and those with diabetes will exceed 700 million worldwide (3). Chronic hyperglycemia is a well-established risk factor for adverse outcomes, including macrovascular complications such as peripheral artery disease, coronary heart disease, and stroke, as well as microvascular complications such as diabetic kidney disease, neuropathy, and retinopathy (4). There are significant differences in the trajectory of glucose metabolism risk in women over the life course. At a young age, dysglycemia is less common in women; however, with increasing age—and especially after menopause, when risk factors for dysglycemia accumulate—this difference diminishes, and the risk of diabetes in women increases (5).

Dyslipidaemia is a common feature of diabetes and is one of the important risk factors for abnormal blood glucose (6). Abnormalities in lipid metabolism are correlated with pancreatic β-cell dysfunction and insulin resistance (IR), contributing to the pathogenesis of diabetes (7). Diabetic patients often present with an atherogenic lipid profile, including abnormalities in blood lipids and lipoproteins (8). Moreover, menopause exerts profound impacts on the social, physiological, and psychological health of women. Postmenopausal women typically exhibit a lipid profile that is considered disadvantageous for health compared with premenopausal women, characterized by elevated levels of low-density lipoprotein cholesterol (LDL-C), triglycerides (TG), and total cholesterol (TC) (9). The decline in ovarian function and hormonal imbalance during menopause can promote abdominal obesity and central visceral fat accumulation, a pattern linked to insulin resistance in non-adipose tissues and organs, significantly increasing the risk of diabetes among postmenopausal women (10). As traditional lipid parameters, TC, TG, high-density lipoprotein cholesterol (HDL-C) and LDL-C are the most commonly used biomarkers to predict diabetes and prediabetes (11–13). However, certain nontraditional lipid measures, such as the the triglyceride glucose index (TyG), TyG-body mass index (TyG-BMI), and TyG-waist-to-height ratio (TyG-WHtR), have been reported to significantly outperform traditional lipid indices in predicting abnormal glucose tolerance, mainly because they integrate multiple lipid profiles to enable a comprehensive assessment of blood glucose levels (14–17). The triglyceride-high density lipoprotein cholesterol-glucose body index (TyHGB) was originally developed and validated by Xu et al. (18) using multivariate linear regression analysis to optimize the assessment of insulin resistance in the context of gestational diabetes mellitus (GDM). This single metric encapsulates the key dimensions of dyslipidemia, dysglycemia, and adiposity.

Given the shared metabolic underpinnings of prediabetes and diabetes in postmenopausal women and gestational diabetes mellitus—particularly with respect to insulin resistance, dyslipidemia, and chronic inflammation—we hypothesized that TyHGB may similarly serve as a powerful marker for prediabetes and diabetes in this population. However, to date, no study has investigated the relationship between TyHGB and the risk of prediabetes and diabetes in postmenopausal women. Therefore, the objectives of the present study were to evaluate the associations between lipid parameters and abnormal blood glucose and to identify the efficacy of lipid predictors in screening for undiagnosed diabetes and prediabetes among community -dwelling postmenopausal women in Dalian.

## Materials and methods

**Figure 1:**
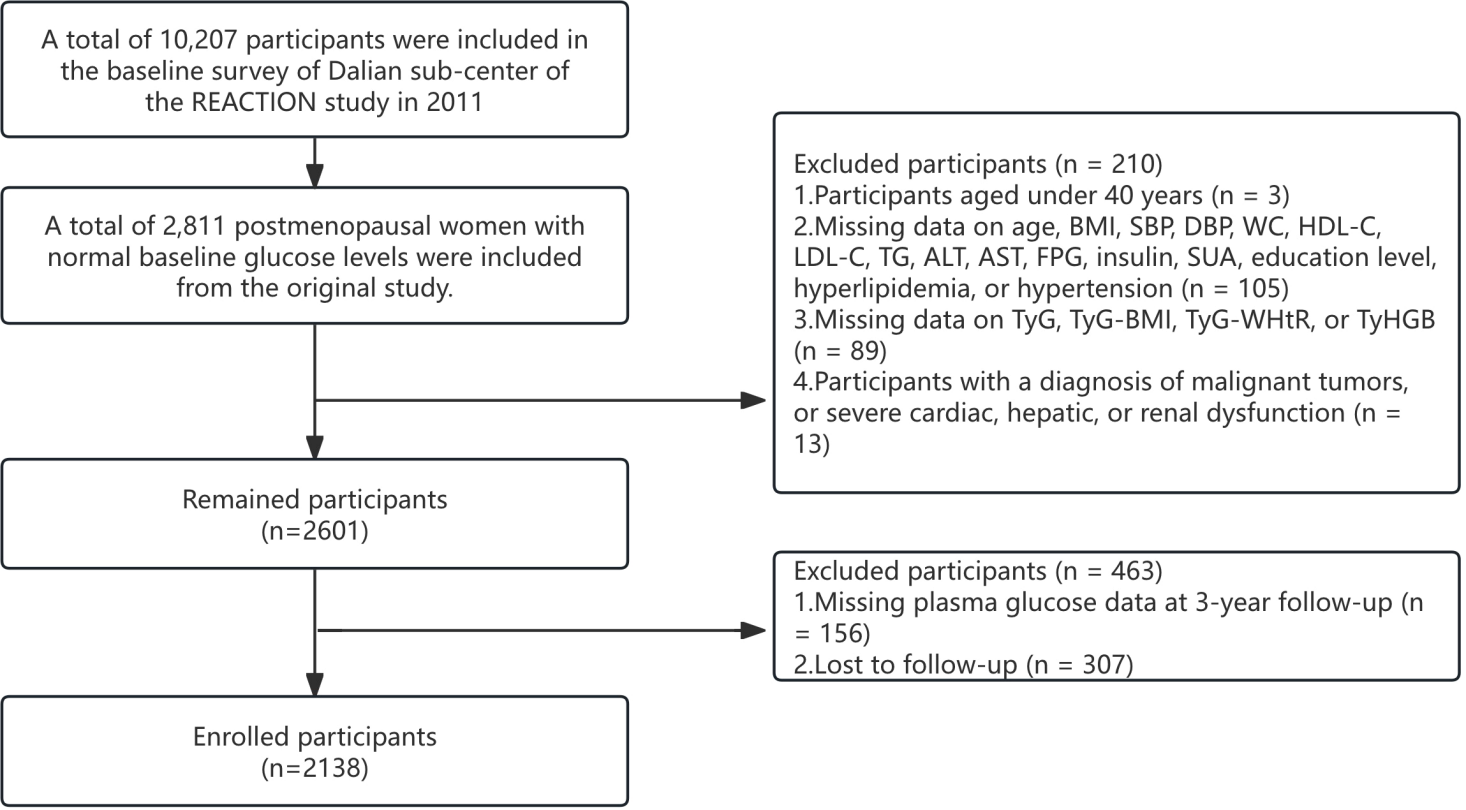
Screening flowchart.

This study initially considered 10,207 community residents aged over 40 from Dalian, who were enrolled in the REACTION study (Risk Evaluation of Cancers in Chinese Diabetic Individuals: A Longitudinal Study)—a project investigating the risk of malignancies in type 2 diabetes mellitus (T2DM) patients in China between August and December 2011, with follow-up conducted in 2014. As the follow-up was performed at the same two time points for all participants, the median follow-up duration was consistently three years. The inclusion criterion was normal baseline glucose levels in postmenopausal women. Exclusion criteria comprised incomplete data, diagnosis of malignant tumors, severe cardiac, hepatic, or renal dysfunction, and loss to follow-up. After applying these criteria, a total of 2,138 participants were included in the final analysis. The detailed screening process is illustrated in **Figure 1**. Ethical approval was obtained from the REACTION Research Ethics Committee (Approval No. 2011 Linlun Review No. 14), and written informed consent was provided by all participants. All procedures were performed in accordance with relevant guidelines and regulations.

### Glucose tolerance status

A standard 75-g oral glucose tolerance test (OGTT) was used to define glucose tolerance status. The test was conducted in the morning after an overnight fast of at least 8 hours. Participants were instructed to avoid strenuous exercise on the preceding day and to refrain from smoking before and during the test. Individuals with medical contraindications did not undergo the OGTT. Venous blood samples were collected at fasting and 2 hours after the ingestion of 75 g of anhydrous glucose for plasma glucose measurement.

### Definition of diabetes, prediabetes and normal glucose tolerance

Diabetes and prediabetes were identified based on any of the following criteria during follow-up: (1) The 1999 World Health Organization (WHO) diagnostic criteria for diabetes and prediabetes were adopted in this study (19). Normal glucose tolerance (NGT) was defined as a fasting plasma glucose < 6.1 mmol/L and a 2-hour post-load glucose < 7.8 mmol/L. Prediabetes was defined as the presence of impaired fasting glucose (IFG; fasting plasma glucose 6.1–6.9 mmol/L), impaired glucose tolerance (IGT; 2-hour post-load glucose 7.8–11.0 mmol/L), or a combination of both. Diabetes was defined as a fasting plasma glucose ≥ 7.0 mmol/L or a 2-hour post-load glucose ≥ 11.1 mmol/L. (2) Self-reported physician diagnosis of diabetes or prediabetes, which was subsequently confirmed by OGTT.

### Calculation of indices

Body Mass Index (BMI) = body mass (kg)/height2(m2);
WHtR = waist circumference (WC)/height;
TyHGB = TG/HDL-C + 0.7*fasting plasma glucose (FPG) (mmol/L) + 0.1*BMI (kg/m2);
TyG = ln [TG (mg/dl) × FPG (mg/dl)/2];
TyG-WHtR = TyG × WHtR;
TyG-BMI = TyG × BMI.

### Covariates

At the study center visit, participants completed self-administered questionnaires and undertook face-to-face interviews with trained nurses to collect baseline data, including age, sex, marital status, education level, smoking status, alcohol consumption, and histories of hypertension, diabetes, hyperlipidemia, and coronary heart disease (CHD). Additionally, all participants underwent a standardized physical examination that included measurements of body mass, height, WC, and blood pressure. An OGTT was administered, and blood samples were collected for laboratory analysis. The laboratory measurements comprised TG, HDL-C, LDL-C, FPG, serum creatinine (SCr), serum uric acid (SUA), aspartate aminotransferase (AST), and alanine aminotransferase (ALT). Based on these data, the following indices were calculated: TyG, TyG-BMI, TyG-WHtR, and TyHGB.

### Statistical analysis

Normality was assessed for all continuous variables. Non-normally distributed data were analyzed using the Mann–Whitney U test and are presented as medians with interquartile ranges (Q25, Q75). Categorical variables were summarized as frequencies and percentages (n%), and group comparisons were performed using the chi-square test. Multivariable logistic regression was employed to evaluate the association between TyHGB and both prediabetes and diabetes. Given the absence of established cut-off values, TyHGB was analyzed by quartiles. Three regression models were constructed: Model 1 (unadjusted), Model 2 (adjusted for age), and Model 3 (further adjusted for age, BMI, systolic blood pressure (SBP), diastolic blood pressure (DBP), WC, HDL-C, LDL-C, TG, ALT, AST, FPG, insulin, SUA, education level, hyperlipidemia, and hypertension). The Cochran–Armitage trend test was used to assess trends across TyHGB quartiles. Dose–response relationships were examined using restricted cubic splines (RCS). Subgroup analyses were conducted to evaluate potential effect modification by covariates (e.g., age, hypertension, hyperlipidemia, and CHD), with statistical significance for interaction terms set at *P* < 0.05. The predictive ability of TyHGB for prediabetes and diabetes was assessed using receiver operating characteristic (ROC) curve analysis, reporting the area under the curve (AUC), sensitivity, and specificity. Concurrently, we used a calibration curve to evaluate the consistency between predicted probability and actual occurrence rate. In addition, decision curve analysis (DCA) assesses the model’s clinical net benefit. All analyses were performed using EmpowerStats (version 4.2) and R software (version 4.3.2). A two-sided *P*-value < 0.05 was considered statistically significant.

## Result

### Baseline characteristics

A total of 2,138 postmenopausal women with normal baseline glucose levels were included in the study. During follow-up, 598 developed prediabetes and 124 developed diabetes. Compared to the normoglycemic group, participants with prediabetes were significantly older and had higher levels of SBP, DBP, BMI, WC, TG, FPG, insulin, ALT, SCr, SUA, TyG, TyG-BMI, TyG-WHtR, and TyHGB (all *P* < 0.05). They also exhibited a higher prevalence of hypertension and hyperlipidemia (both *P* < 0.01), alongside lower levels of HDL-C and educational attainment (both *P* < 0.01). Similarly, the diabetes group was significantly older and had higher levels of SBP, DBP, BMI, WC, LDL-C, TG, FPG, insulin, ALT, SUA, TyG, TyG-BMI, TyG-WHtR, and TyHGB (all *P* < 0.01) than the normoglycemic group. They were also more likely to have CHD (*P* < 0.05) and had lower levels of HDL-C and education (**Table 1**).

**Table 1:** Clinical characteristics of study participants.

Characteristics	Control	Prediabetes	Diabetes	*P*-value
	(N = 1416)	(N = 598)	(N = 124)	
Age (years)	57.04 (53.79, 60.81)	58.80 (54.72, 64.72)	59.11 (55.75, 65.04)	<0.01
SBP (mmHg)	134.83 (122.58, 149.00)	142.00 (129.33, 154.92)	148.17 (131.50, 164.17)	<0.01
DBP (mmHg)	77.67 (70.67, 85.33)	80.33 (72.67, 87.00)	81.33 (73.58, 89.67)	<0.01
BMI (kg/m2)	24.77 (22.58, 27.06)	25.83 (23.65, 28.12)	26.69 (24.68, 29.03)	<0.01
WC (cm)	87.00 (80.00, 94.00)	90.00 (84.00, 96.00)	93.00 (85.75, 99.00)	<0.01
HDL-C (mmol/L)	1.47 (1.28, 1.69)	1.38 (1.21, 1.59)	1.37 (1.22, 1.57)	<0.01
LDL-C (mmol/L)	3.33 (2.79, 3.90)	3.42 (2.86, 3.98)	3.59 (3.09, 4.04)	<0.01
TG (mmol/L)	1.18 (0.87, 1.65)	1.48 (1.06, 1.98)	1.59 (1.21, 2.30)	<0.01
FPG (mmol/L)	5.41 (5.14, 5.72)	5.71 (5.40, 6.08)	5.96 (5.54, 6.35)	<0.01
Insulin	7.05 (5.30, 9.80)	8.55 (6.40, 11.60)	9.60 (7.00, 13.55)	<0.01
SCr (μmol/L)	61.10 (56.70, 66.60)	61.80 (57.40, 67.40)	61.05 (57.27, 65.43)	0.07
SUA (μmol/L)	280.00 (247.00, 320.00)	299.00 (263.00, 338.00)	314.50 (272.00, 358.75)	<0.01
ALT (U/L)	15.00 (12.00, 20.00)	17.00 (13.00, 23.00)	19.00 (14.00, 27.00)	<0.01
AST (U/L)	21.00 (18.00, 25.00)	21.00 (19.00, 26.00)	22.50 (19.00, 27.25)	0.02
TyG	8.54 (8.21, 8.88)	8.81 (8.49, 9.09)	8.91 (8.62, 9.30)	<0.01
TyG-BMI	212.58 (190.35, 236.98)	227.99 (204.89, 251.37)	239.08 (221.05, 265.44)	<0.01
TyG-WHtR	4.66 (4.27, 5.17)	5.04 (4.64, 5.47)	5.34 (4.89, 5.75)	<0.01
TyHGB	7.16 (6.62, 7.80)	7.75 (7.21, 8.41)	8.06 (7.61, 8.72)	<0.01
Marriage (%)				0.52
Married	1237 (87.36%)	529 (88.46%)	104 (83.87%)	
Single, divorced or widowed	174 (12.29%)	66 (11.04%)	20 (16.13%)	
Other	5 (0.35%)	3 (0.50%)	0 (0.00%)	
Education (%)				<0.01
Primary school and lower	150 (10.59%)	96 (16.05%)	24 (19.35%)	
middle school	665 (46.96%)	248 (41.47%)	63 (50.81%)	
high school	601 (42.44%)	254 (42.47%)	37 (29.84%)	
CHD (%)				0.05
Yes	54 (3.81%)	31 (5.18%)	10 (8.06%)	
No	1362 (96.19%)	567 (94.82%)	114 (91.94%)	
Hyperlipidemia (%)				0.02
Yes	109 (7.70%)	68 (11.37%)	9 (7.26%)	
No	1307 (92.30%)	530 (88.63%)	115 (92.74%)	
Hypertension (%)				<0.01
Yes	214 (15.11%)	129 (21.57%)	25 (20.16%)	
No	1202 (84.89%)	469 (78.43%)	99 (79.84%)	
Smoking (%)				0.83
No	1385 (98.23%)	588 (98.49%)	122 (99.19%)	
Occasionally	11 (0.78%)	5 (0.84%)	0 (0.00%)	
Frequently	14 (0.99%)	4 (0.67%)	1 (0.81%)	
Drinking (%)				0.43
No	1235 (87.59%)	526 (88.70%)	102 (82.93%)	
Occasionally	159 (11.28%)	62 (10.46%)	20 (16.26%)	
Frequently	16 (1.13%)	5 (0.84%)	1 (0.81%)	

Data are expressed as median (P25, P75) or number (percent). Abbreviation: SBP, systolic blood pressure; DBP, diastolic blood pressure; BMI, body mass index; WC, waist circumference; HDL-C, high-density lipoprotein cholesterol; LDL-C, low-density lipoprotein cholesterol; TG, triacylglycerol; FPG, fast plasma glucose; SCr, serum creatinine; SUA, blood uric acid; ALT, alanine aminotransferase; AST, aspartate aminotransferase; TyG, triglyceride glucose; TyG-BMI, TyG-body mass index; TyG-WHtR, TyG-waist-to-height ratio; TyHGB, triglyceride-high density lipoprotein cholesterol-glucose body index; CHD, coronary heart disease;.

### Association of the TyHGB with prediabetes and diabetes

The results of the multivariate analyses examining the association between the TyHGB index and the incidence of prediabetes and diabetes are presented in **Table 2**. After full adjustment for age, BMI, SBP, DBP, WC, HDL-C, LDL-C, TG, ALT, AST, FPG, insulin, SUA, education level, hyperlipidemia, and hypertension, the TyHGB index was positively associated with an increased risk of both prediabetes (OR = 1.48; 95% CI: 1.32–1.66) and diabetes (OR = 1.57; 95% CI: 1.33–1.84). To further assess the impact of TyHGB levels, the index was categorized into quartiles. For prediabetes, compared to the lowest quartile (Q1), the adjusted odds ratios for Q2, Q3, and Q4 were 2.12 (95% CI: 1.51–2.99), 3.18 (95% CI: 2.23–4.53), and 4.45 (95% CI: 3.03–6.53), respectively. Similarly, for diabetes, the highest quartiles showed a significantly elevated risk, with an OR of 2.64 (95% CI: 1.16–6.00) for Q3 and 6.59 (95% CI: 2.90–14.97) for Q4, compared to Q1.

**Table 2:** Association of TyHGB with prediabetes and diabetes in multivariable logistic regression.

	Model 1		Model 2		Model 3	
	OR (95%CI)	*P*-value	OR (95% CI)	*P*-value	OR (95% CI)	*P*-value
Prediabetes						
TyHGB	1.70 (1.54, 1.88)	<0.01	1.65 (1.50, 1.83)	<0.01	1.48 (1.32, 1.66)	<0.01
Q1	1 (Ref)		1 (Ref)		1 (Ref)	
Q2	2.51 (1.81, 3.47)	<0.01	2.35 (1.69, 3.26)	<0.01	2.13 (1.51, 3.00)	<0.01
Q3	4.00 (2.91, 5.49)	<0.01	3.71 (2.70, 5.11)	<0.01	3.17 (2.23, 4.52)	<0.01
Q4	6.11 (4.45, 8.38)	<0.01	5.59 (4.06, 7.70)	<0.01	4.42 (3.01, 6.50)	<0.01
P-trend	<0.01		<0.01		<0.01	
Diabetes						
	Model 1		Model 2		Model 3	
	OR (95%CI)	*P*-value	OR (95% CI)	*P*-value	OR (95% CI)	*P*-value
TyHGB	1.80 (1.55, 2.10)	<0.01	1.78 (1.53, 2.07)	<0.01	1.55 (1.31, 1.83)	<0.01
Q1	1 (Ref)		1 (Ref)		1 (Ref)	
Q2	2.28 (1.00, 5.17)	0.04	2.07 (0.91, 4.70)	0.08	1.50 (0.64, 3.55)	0.35
Q3	5.11 (2.40, 10.84)	<0.01	4.54 (2.13, 9.69)	<0.01	2.63 (1.15, 6.01)	0.02
Q4	13.42 (6.57, 27.37)	<0.01	11.94 (5.83, 24.45)	<0.01	6.53 (2.86, 14.87)	<0.01
P-trend	<0.01	<0.01	<0.01	<0.01	<0.01	

Model 1: no adjustment.

Model 2: adjusted for age.

Model 3: adjusted for age, SBP, DBP, WC, LDL-C, ALT, AST, insulin, SUA, education level, hyperlipidemia, and hypertension

### Dose-response relationship of TyHGB with prediabetes and diabetes

**Figure 2:**
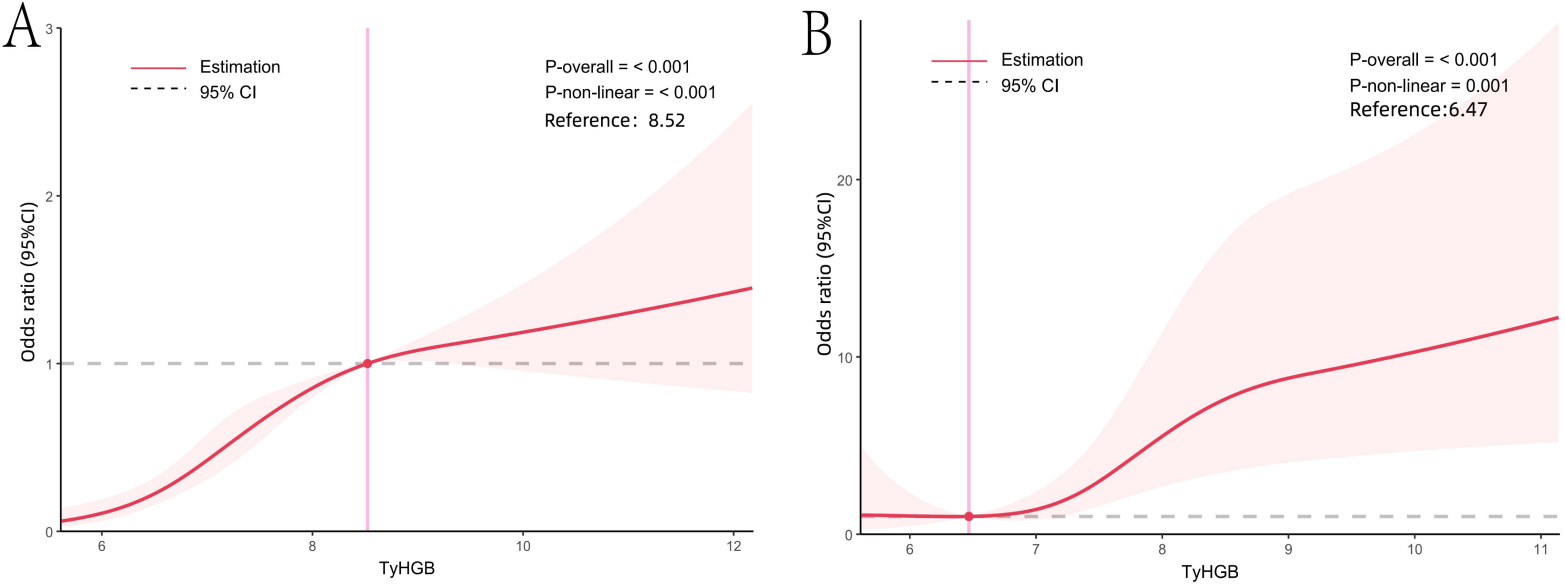
The dose–response relationship of TyHGB with prediabetes **(A)** and diabetes **(B)**. Graphs show OR for prediabetes and diabetes adjusted for age, SBP, DBP, WC, LDL-C, ALT, AST, insulin, SUA, education level, hyperlipidemia, and hypertension. Data were fitted by multivariate logistic regression models. Solid lines indicate OR, and shadow shapes indicate 95% CIs.

**Figure 2** illustrates the dose-response relationships of TyHGB with prediabetes and diabetes, analyzed using RCS. After adjustment for multiple covariates, a higher TyHGB level was associated with an increased risk of both disorders. A significant non-linear relationship was identified for prediabetes (*P* for non-linearity < 0.01), with a threshold value observed at 8.52. Below this inflection point, the risk of prediabetes increased steeply with rising TyHGB; above the threshold, the curve plateaued, showing a more gradual elevation. Similarly, a significant non-linear association was observed between TyHGB and diabetes risk (*P* for non-linearity < 0.01), with a threshold at 6.47. In contrast to the pattern observed for prediabetes, the risk of diabetes remained relatively stable below the inflection point, after which it increased markedly with further TyHGB elevation.

### Subgroup analysis

**Figure 3:**
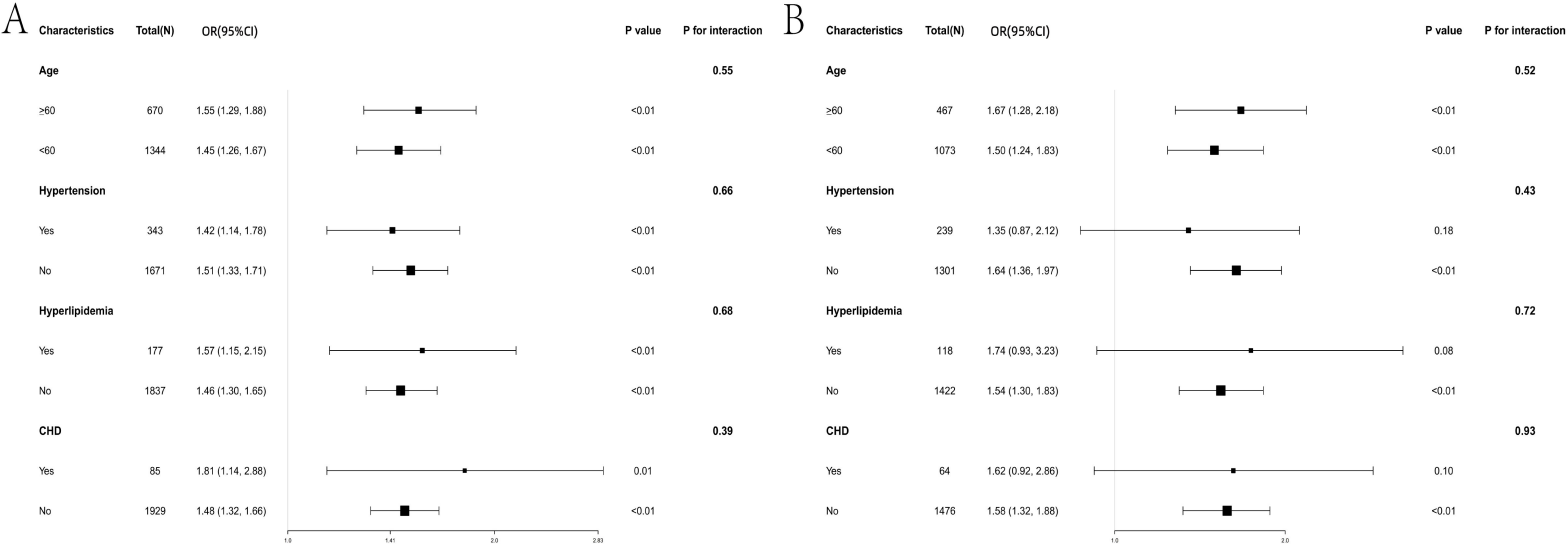
Subgroup analysis of the association between TyHGB and prediabetes **(A)** and diabetes **(B)**. Subgroup analysis of the association between TyHGB and prediabetes and diabetes, stratified by age, hypertension, and CHD. Abbreviation: TyHGB, triglyceride-high density lipoprotein cholesterol-glucose body index; CHD, coronary heart disease; OR, odds ratio.

Furthermore, subgroup analyses were conducted to assess the consistency of the association between TyHGB and dysglycemia (prediabetes and diabetes) across various clinical factors (**Figure 3**). Stratification was performed by age, hypertension, hyperlipidemia, and CHD. The results revealed that a higher TyHGB level was significantly associated with an increased risk of dysglycemia, and this association was consistent across all subgroups (all *P* for interaction > 0.05).

### Predictive value of TyHGB for prediabetes and diabetes

**Figure 4:**
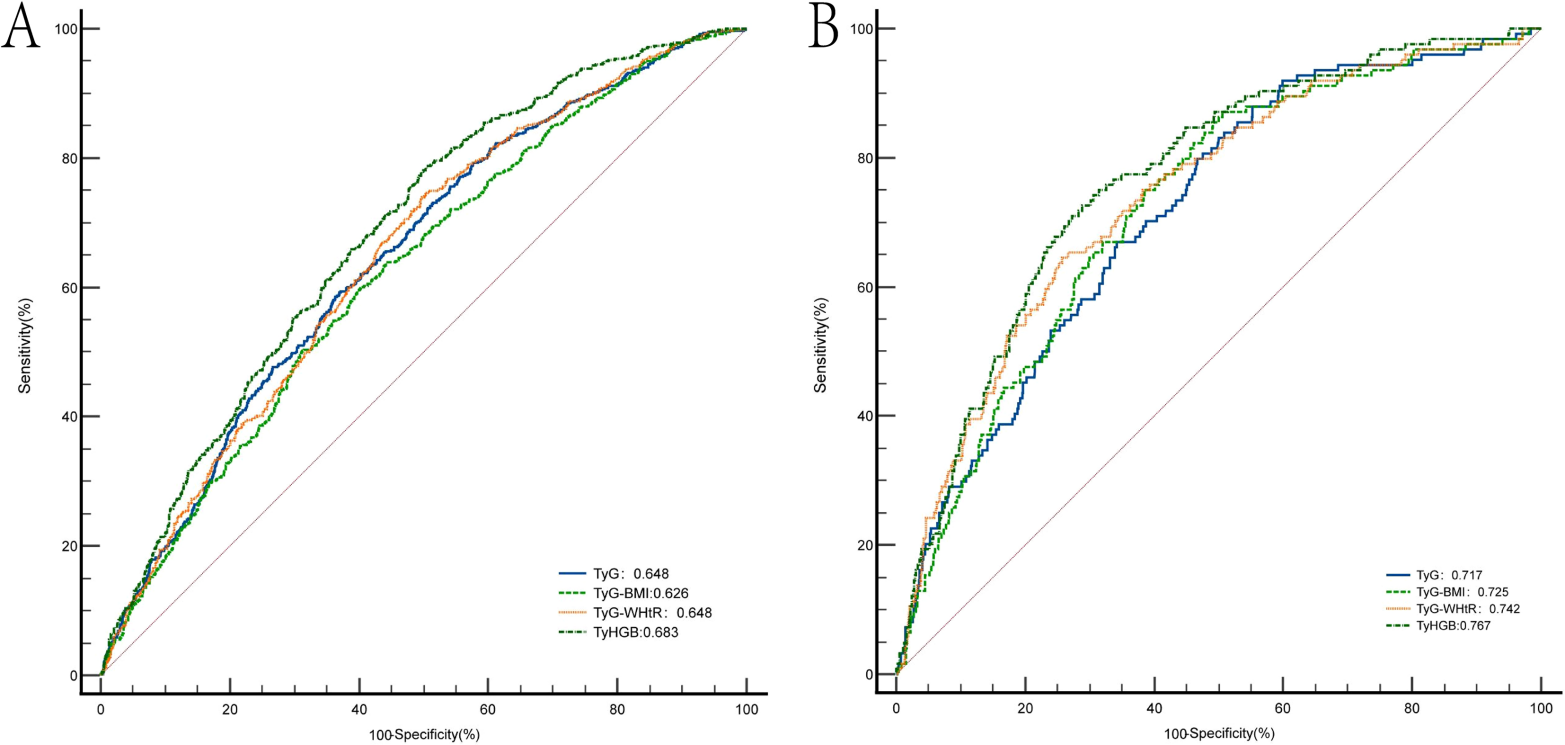
ROC curves and the AUC values of TyHGB in diagnosing prediabetes **(A)** and diabetes **(B)**.

**Figure 5:**
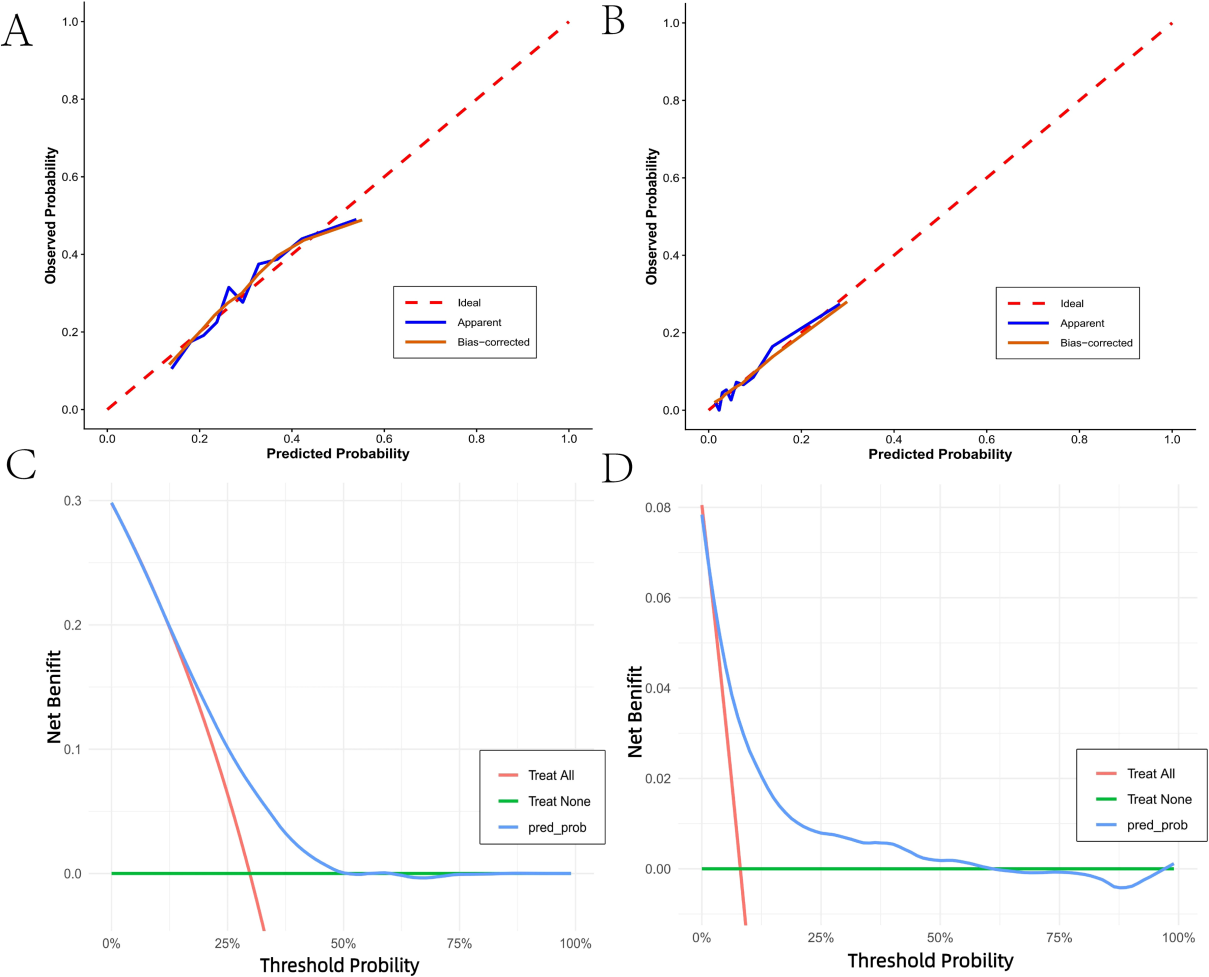
Calibration plot for the prediabetes **(A)** and diabetes **(B)**, DCA curves for the prediabetes **(C)** and diabetes **(D)**.

The ROC curves for the different indices are shown in **Figure 4**. The corresponding AUC values, optimal cutoff points, sensitivity, specificity, and Youden’s index are detailed in **Table 3**. For prediabetes, the TyHGB index demonstrated the highest diagnostic performance (AUC = 0.683; 95% CI, 0.658-0.707), followed by the TyG (AUC = 0.648; 95% CI, 0.622-0.674), TyG-WHtR (AUC = 0.648; 95% CI, 0.623-0.674), and TyG-BMI index (AUC = 0.626; 95% CI, 0.600-0.652). The DeLong test further revealed that the AUC of TyHGB was significantly higher than that of TyG, TyG-WHtR, and TyG-BMI (Z = 4.154, 3.578, 6.962; all *P* < 0.05). The optimal cutoff values were 7.146 for TyHGB, 8.701 for TyG, 4.670 for TyG-WHtR, and 220.445 for TyG-BMI. For diabetes, the TyHGB index again showed the highest AUC (0.767; 95% CI, 0.726-0.808), higher than TyG-WHtR (AUC = 0.742; 95% CI, 0.700-0.787), TyG-BMI (AUC = 0.725; 95% CI, 0.682-0.769), and TyG (AUC = 0.717; 95% CI, 0.672-0.761). The DeLong test further revealed that the AUC of TyHGB was significantly higher than that of TyG, and TyG-BMI (Z = 3.965, 3.078; all *P* < 0.05). Notably, no significant difference in AUCs was observed between TyHGB and TyG-WHtR (*P* > 0.05). The respective optimal cutoff values were 7.688 for TyHGB, 5.147 for TyG-WHtR, 212.076 for TyG-BMI, and 8.592 for TyG. Furthermore, the calibration curves showed good agreement between predicted and observed probabilities for both prediabetes (**Figure 5A**) and diabetes (**Figure 5B**), indicating satisfactory calibration of the fully adjusted model. Moreover, the DCA showed that the fully adjusted model yielded a higher net benefit than both the “treat all” and “treat none” strategies across a wide range of threshold probabilities for both prediabetes (**Figure 5C**) and diabetes (**Figure 5D**).

**Table 3:** Performance of TyHGB to evaluate prediabetes and diabetes and optimal critical points.

Variable	AUC	95% CI	Sensitivity	Specificity	Youden index	optimal cutoff points	z statistic	*P*-value
Prediabetes								
TyHGB	0.683	0.658-0.707	0.496	0.788	0.283	7.146	Reference	Reference
TyG	0.648	0.622-0.674	0.631	0.592	0.223	8.701	4.154	<0.001
TyG-WHtR	0.648	0.623-0.674	0.503	0.739	0.242	4.670	3.578	<0.001
TyG-BMI	0.626	0.600-0.652	0.603	0.595	0.198	220.445	6.962	<0.001
Diabetes								
TyHGB	0.767	0.726-0.808	0.712	0.726	0.438	7.688	Reference	Reference
TyG-WHtR	0.742	0.700-0.787	0.743	0.645	0.388	5.147	1.373	0.170
TyG-BMI	0.725	0.682-0.769	0.494	0.871	0.365	212.076	3.078	0.002
TyG	0.717	0.672-0.761	0.533	0.798	0.331	8.592	3.965	<0.001

AUC, area under the curve; 95% CI, 95% confidence interval.

## Discussion

This longitudinal study, based on a cohort of middle-aged and elderly postmenopausal women in Dalian, China, is the first to investigate the association between the TyHGB and the risk of developing prediabetes and diabetes in this population. During the three-year follow-up period, among 2,138 postmenopausal women with normal baseline glucose levels, 598 progressed to prediabetes and 124 developed diabetes. Our findings demonstrate a significant positive association between TyHGB and the risk of both prediabetes and diabetes, with restricted cubic spline analysis revealing a nonlinear dose-response relationship characterized by distinct patterns for each outcome. For incident prediabetes, the risk increased steeply with rising TyHGB below the inflection point of 8.52, after which the curve showed a more gradual elevation. In contrast, for incident diabetes, the risk remained relatively stable until reaching the inflection point of 6.47, beyond which it increased markedly with further TyHGB elevation. Notably, ROC curve analysis suggested that TyHGB may serve as a superior predictor for identifying postmenopausal women at risk for dysglycemia compared to traditional indicators, especially for diabetes.

Postmenopausal women represent a high-risk population for diabetes due to declining estrogen levels, which are often accompanied by exacerbated lipid metabolism disorders and insulin resistance (20, 21). A previous study has investigated the association between the TyHGB index and the risk of GDM. The findings indicate that TyHGB is significantly associated with the risk of GDM. The favorable predictive performance of the TyHGB index suggests its potential utility as a screening and monitoring tool for pregnant women at high risk of GDM (18). However, to date, no studies have assessed TyHGB in relation to the risks of diabetes or prediabetes in postmenopausal women experiencing complex physiological and metabolic changes. Our study fills a critical gap in the literature. We observed a significant positive association between TyHGB levels and the prevalence of diabetes or prediabetes in postmenopausal women. A significant positive trend was observed across increasing TyHGB quartiles, with participants in the highest quartile had a 5.59-fold increased risk of diabetes compared to those in the lowest quartile, and a 3.45-fold increased risk of prediabetes.

The TyHGB index integrates parameters related to glucose and lipid metabolism as well as obesity, which may explain its superiority in predicting dysglycemia risk. Several biological mechanisms may explain the association between TyHGB and dysglycemia risk in postmenopausal women. First, the decline in ovarian endocrine function after menopause leads to reduced estrogen secretion. It is currently understood that estrogen increases LDL receptors on hepatocytes while decreasing HDL receptors. This promotes the hepatic clearance of chylomicrons, accelerates bile acid secretion, and facilitates the elimination of cholesterol from the body. The net effect is an improvement in lipid profile composition, characterized by elevated HDL levels and reduced LDL, TC, and TG levels (22). These metabolic changes induced by estrogen deficiency not only lead to lipotoxicity but also disrupt organelle function, resulting in the release of excessive reactive oxygen species (ROS) and pro-inflammatory factors, which then trigger a systemic inflammatory response. In addition, these pathological changes can also interfere with the normal role of insulin in the insulin signaling pathway, eventually leading to a state of insulin resistance (23–27). Second, decreased estrogen levels result in lower sex hormone-binding globulin (SHBG) synthesis, a change that has been implicated in the heightened risk of insulin resistance following menopause (28). Clinical studies have confirmed that SHBG levels are negatively correlated with insulin resistance in postmenopausal women, whereas no such correlation has been observed in premenopausal women (29). After menopause, total androgen levels decrease. However, due to the reduction in SHBG, the levels of free and bioavailable androgens increase relatively. This relative increase in androgen activity is considered to be associated with insulin resistance (30, 31). Additionally, after menopause, fat distribution shifts from the gyneoid to the android pattern, resulting in visceral fat accumulation (32). This central distribution of body fat exacerbates the risk of insulin resistance (33). Insulin resistance is the core pathological mechanism of diabetes and prediabetes; therefore, it can be speculated that elevated TyHGB levels are associated with an increased risk of diabetes and prediabetes (34). Of note, restricted cubic spline analyses in the present study showed that the associations between TyHGB and the risks of incident prediabetes and diabetes displayed distinct non-linear threshold profiles. The risk inflection point for prediabetes (8.52) was higher than that for diabetes (6.47). This finding does not indicate that individuals with prediabetes have higher absolute TyHGB levels; instead, it reflects stage-dependent differences in risk sensitivity and the pattern of risk acceleration in response to elevated TyHGB. For prediabetes, the risk increased steeply at relatively low TyHGB levels, suggesting that mild elevations in TyHGB are sufficient to induce impaired glucose metabolism. Conversely, for incident diabetes, the risk remained relatively stable at low TyHGB levels and increased markedly only after exceeding the inflection point of 6.47, indicating that higher TyHGB levels serve as a key driver for the development of overt diabetes. Collectively, these results highlight a stage-specific threshold effect of TyHGB on dysglycemia. However, these inflection points were derived from a single cohort and should be considered hypothesis-generating rather than clinically actionable cutoffs. External validation in independent populations is essential before these thresholds can be applied to clinical practice or intervention decisions. Future studies are warranted to confirm the reproducibility and biological meaning of these stage-specific thresholds.

The consistent association between TyHGB and diabetes risk across subgroups defined by age, hypertension, hyperlipidemia, and coronary heart disease (all *P* for interaction > 0.05) supports the robustness of our findings. This indicates that TyHGB may serve as a reliable predictor for diabetes risk in postmenopausal women, regardless of these clinical conditions.

ROC curve analysis showed that, in postmenopausal women, TyHGB had the highest AUC among all indices for predicting prediabetes, but its predictive value was modest (AUC < 0.7); whereas for predicting diabetes, its predictive performance was comparable to that of TyG-WHtR and superior to that of TyG and TyG-BMI. These results indicate that TyHGB has potential application value in predicting diabetes in postmenopausal women. The calibration curves showed good agreement, and the decision curve analysis yielded higher net benefit across a wide range of threshold probabilities, confirming satisfactory calibration and clinical utility. Consistently, previous studies have established the predictive value of TyG in general populations or specific subgroups, including individuals with gestational diabetes mellitus (35–38). A longitudinal study of 15,012 prediabetic adults (2010–2016) found that both the TyG index and TG/HDL-C ratio were significantly associated with diabetes progression, and the TyG index showed superior predictive accuracy (39). Another study using data from the China Health and Retirement Longitudinal Study (CHARLS), which included 6,258 participants aged ≥45 years, compared the TyG index, its derived parameters, visceral adiposity index (VAI), lipid accumulation product (LAP), and triglyceride-to-HDL cholesterol ratio (TG/HDL-C) for their associations with diabetes risk. The findings revealed that TyG-WHtR served as a clinically effective marker for identifying diabetes risk in the NFG group, whereas TyG-BMI was effective for predicting diabetes in the IFG group (40). Nevertheless, these indices were originally developed and optimized in general or metabolically homogeneous populations, and their performance in populations with unique physiological characteristics—such as postmenopausal women—remains incompletely characterized. Critically, existing TyG-related indices primarily emphasize the interplay between triglycerides and glucose, yet they do not adequately incorporate HDL cholesterol, a key lipid parameter that is profoundly affected by estrogen decline and plays an independent protective role against insulin resistance. TyG-BMI and TyG-WHtR introduce adiposity measures but still lack direct adjustment for the lipid profile alterations specific to the menopausal transition, such as the characteristic decrease in HDL-C (41). Consequently, the generalizability of these traditional indices to postmenopausal women may be limited. This discrepancy may be attributable to the unique metabolic characteristics of the postmenopausal state, including estrogen deficiency-induced lipid metabolism disorders, central obesity, and increased insulin resistance (42). In contrast to existing indices, the TyHGB index was specifically designed to integrate glycemic, lipid (including TG and HDL-C), and body composition parameters within a single composite metric. This integrative design allows TyHGB to capture the synergistic effects of dyslipidemia, dysglycemia, and central adiposity, which collectively are associated with insulin resistance and pancreatic β-cell dysfunction. Therefore, although its predictive performance is comparable to that of TyG-WHtR, TyHGB has a superior pathophysiological rationale: it directly reflects the typical combination of metabolic abnormalities in postmenopausal women. This multidimensional integration may provide richer information for clinical interpretation and individualized risk assessment, making it particularly suitable for diabetes risk stratification in this high−risk population.

### Limitations and future directions

Several limitations of this study should be acknowledged, along with corresponding directions for future research. First, the study population was restricted to middle-aged and elderly postmenopausal women from Dalian, China (a single-city cohort), which may limit the generalizability of our findings to other ethnic or geographic populations. Future studies should validate these findings in multicenter cohorts encompassing diverse ethnic and geographic backgrounds. Second, the absence of HbA1c measurements precluded direct comparison with the OGTT-based classification and limited our ability to assess glycemic control more comprehensively. Future studies incorporating HbA1c data are needed to further validate the utility of TyHGB. Third, the relatively short follow-up period of three years may be insufficient to capture long-term trajectories of glycemic deterioration, although it allowed detection of incident dysglycemia. Longitudinal studies with extended follow-up periods are warranted to assess the predictive value of TyHGB for long-term diabetes risk and its complications. Fourth, we lacked comprehensive data on medication use (including glucose-lowering agents and antihypertensive drugs) and hormone replacement therapy, which may influence glucose metabolism and confound the observed associations. Future studies should collect detailed information on these medications to better control for potential confounding. Fifth, despite adjusting for multiple confounders, residual confounding from unmeasured factors such as dietary patterns, physical activity, and family history of diabetes cannot be excluded. Well-designed prospective studies with comprehensive data collection on lifestyle factors and relevant confounders are needed to confirm the independent association between TyHGB and dysglycemia risk. Finally, the TyHGB index was developed and validated only in this single cohort without external validation in an independent population. External validation studies are therefore essential before the TyHGB index can be recommended for clinical use or generalized to other populations.

## Conclusion

In this longitudinal cohort study of middle-aged and elderly postmenopausal women with normal baseline glucose levels, we found that TyHGB was significantly and positively associated with the risk of developing prediabetes and diabetes over a three-year follow-up period. Furthermore, TyHGB may provide effective predictive value for diabetes in postmenopausal women. These findings suggest that TyHGB may serve as a valuable and simple tool for identifying high-risk postmenopausal women who could benefit from targeted preventive interventions. Further validation in diverse populations is warranted.

## Data Availability

The raw data supporting the conclusions of this article will be made available by the authors, without undue reservation.
